# Fertility preservation in patients with uterus didelphys and endometrial carcinoma: a case report

**DOI:** 10.1186/s12905-021-01455-6

**Published:** 2021-08-28

**Authors:** Jiao Yu, Jing Shang, Hongwu Wen, Yang Xu

**Affiliations:** Department of Obstetrics and Gynecology, Peking University First Hospital, Beijing, 100034 China

**Keywords:** Endometrial cancer, Fertility preservation, IHC analysis, Uterus didelphys

## Abstract

**Background:**

Endometrial cancer combining uterus didelphys is quite rare clinically which partially explains that there is no discussion about young patients’ fertility preservation and follow up of tumor outcome.

**Case presentation:**

In this article, we report a case of unilateral endometrial carcinoma found in a young patient with uterus didelphys who was treated with high-efficiency progesterone due to unfinished child-bearing. During the follow-up, the affected uterine endometrium was not reversed by progesterone. So, the patient underwent the abdominal surgery with the left uterus and left fallopian tube resection. We performed three consecutive immunohistochemical studies of the contralateral uterine endometrium to verify the safety of preserving the contralateral uterus and its appendages which preserved her fertility.

**Conclusions:**

Endometrial cancer occurring in patients with uterus didelphys is quite rare in child-bearing age. In this case report, we preserved the patient’s contralateral uterus based on patient’s strong needs and negative IHC analysis of the preserved side uterine endometrium. However, the tumor and fertility outcome require more follow-up.

## Background

Uterus didelphys is a relatively rare type of congenital malformation of female genital tract due to the complete absence of fusion of bilateral paramesonephric tubes during embryonic development [1, 2]. According to the degree of abnormal fusion, uterus didelphys can combine double cervix and(or) double vagina malformations. At most cases, uterus didelphys does not need corrective surgery because of their negative clinical symptoms.

Genitourinary system is originated from mesoderm. The development of mesonephric tube or paramesonephric tube will be impaired with the effects of adverse factors during embryonic development which will lead to abnormalities of urinary system and reproductive system. Therefore, malformations of reproductive system are often accompanied by abnormalities of urinary system [3].

Endometrial carcinoma is one of the most common gynecological malignancies. However, it is extremely rare to have uterus didelphys and endometrial carcinoma in one patient. The standard treatment for endometrial cancer is extra fascial hysterectomy and bilateral adnexectomy, pelvic lymphadenectomy and other biopsy as appropriate [4]. Until now, there are few case reports of uterus didelphys combined with endometrial cancer. Our case reported uterus didelphys with unilateral endometrial carcinoma in a young patient wanting to preserve fertility. We had her contralateral uterus kept which was distinctive and unique compared with published studies.

## Case presentation

The female patient was diagnosed as uterus didelphys at 28 years old when she was doing the transvaginal ultra-sound because of long-term abnormal uterine bleeding as 10–15 days/30 days. The gynecological physical and imaging examination confirmed the diagnosis as “double uterus, double cervix, double vagina”. She was operated hysteroscopy with endometrial polyp removal and segmental curettage in October 2018 for the first time. During the operation, it was found that there was a single polyp in the right cavity and multiple polyps in the left cavity. Postoperative paraffin pathology and immunohistochemistry showed that there was endometrial complex atypical hyperplasia and focal carcinoma (moderately differentiated endometrioid carcinoma) in the left cavity. IHC: ER 90% strong positive, PR 90% strong positive, p53+, Pax8+++, PTEN-, p16-, Ki67 10–30%. No lesion was found in the right uterine endometrium. The patient of childbearing age required conservative treatment which was taking oral megestrol acetate after surgery. In March 2019, hysteroscopy and curettage were reexamined. The postoperative pathology reported that there was mucinous metaplasia of endometrial glands in the left uterine cavity which can be explained because of the medication.

After the operation, the patient continued to take megestrol acetate. In June 2019, the hysteroscopy and segmental curettage were reexamined. Postoperative pathology showed that the endometrium of left uterus was moderately differentiated endometrioid carcinoma. With strong needs for child-bearing, our patient continuously chose to take megestrol acetate and reviewed hysteroscopy and curettage again in October 2019. Postoperative pathology result of left uterine endometrium was still in accordance with endometrioid carcinoma (moderately differentiated). Doctors recommended surgical treatment due to the unsatisfied outcome of conservative drug treatment. However, the patient refused and continued to take megestrol acetate for outpatient follow-up. Things changed in April 2020 when the pelvic enhanced MRI of our patient showed abnormal enhancement of left endometrium which was more obvious than before. In May 2020, she finally underwent the abdominal surgery with the left uterus and left fallopian tube resection. There were two spindle shaped uterus and accessories in bilateral iliac fossa. During the surgery, we opened the left ureteral tunnel, pushed down the bladder to 3 cm below the external orifice of the cervix and blocked the left uterine artery above it. The left utero-sacral ligament was cut and sewed 2 cm beside the uterus. The vaginal septum was cut open. We removed the left uterus along the space between the two cervixes. The left uterine endometrium was thick and crisp according to the postoperatively dissected specimen. The pathological findings were consistent with moderately differentiated endometrioid carcinoma with changes after treatment in the left uterus. The molecular and gene detection of the specimen showed that it may belong to the molecular type of MMR deficiency type which belongs to sporadic endometrial carcinoma and does not belong to Lynch syndrome.

The patient recovered well after operation. Combined with postoperative pathology, the patient was diagnosed as stage IA of moderately differentiated endometrial carcinoma in the left uterus. No abnormalities were found in the postoperative reexamination during which she has been trying to get pregnant in fertility center.

## Discussion and conclusions

Female reproductive tract parenchyma develops from epithelia of the Mullerian Duct and urogenital sinus [5]. The Mullerian duct is derived from intermediate mesoderm and composed of epithelial and mesenchymal cells [1]. The development of the Mullerian ducts includes specification, invagination and elongation. Around gestational week 8, the Mullerian ducts’ terminal ends join together. A midline epithelial septum separates the lumen of two adjacent Mullerian ducts temporarily. During the 9th week, the midline septum disappears largely resulting in the formation of the midline uterovaginal canal. At about 12 weeks gestation, the basement membranes disappearing makes the uterus created. At about 14–15 weeks, gland formation in the uterine corpus and uterine cervix initially appears. The primary glands are found distant from the uterine fundus which proves that the first forming glands are possible to be cervical glands. The uterine and cervical glands which are initially lined by a simple columnar epithelium are elongated and branched within the stroma by 20 weeks [6, 7]. Based on the embryological observations, it can be speculated that the two uterine cavities of double uterus have the same risk of pathological changes.

We reviewed published articles about uterus didelphys combining endometrial cancer in PubMed from 1990 to 2020 [8–22]. There are 15 cases included, the age of onset ranged from 33 to 75 years old. Among them, 4 cases [11, 12, 15, 16] were less than 45 years old. Only 1 case had finished child-bearing before operation [12], 3 cases had staged operation, and only 1 case had bilateral adnexa reserved [11]. Many guidelines and experts achieved consensus on fertility preservation therapy for endometrial cancer at home and abroad. There are no discussion about the preservation of fertility function in young patients with uterus didelphys and endometrial carcinoma due to the rare occurrence.

In this case, an infertile woman with uterus didelphys was diagnosed as endometrial cancer in the left uterine cavity at the age of 28 years. The patient's endometrium did not achieve reversal after the classic high-efficiency progesterone treatment which led to the surgery to remove the left uterus. According to the embryological observations, uterus didelphys is due to the failure of fusion of the Mullerian ducts to form the uterus. Whether to keep the contralateral uterus is worthy of more discussion and attention.

By reviewing the previous 15 cases of double uterine malformation with endometrial cancer, we concluded that 7 cases (7/15) occurred in the right uterus with cancer staging from Ia–II and differentiation types from G1–G3. Among the 7 cases, only 1 case combined with atypical hyperplasia in the left endometrium. 4 cases (4/15) occurred in the left uterus with cancer staging from Ia-IIIc and differentiation type from G1 to G3, 1 of 4 cases was associated with atypical hyperplasia in the right endometrium. 4 cases (4/15) occurred in bilateral uterus with 1 case in stage IA (differentiation type G1), 1 case in stage II (differentiation type G2), 1 case in stage IIIA (differentiation type G1), and 1 case in stage IV (differentiation type G3).

From the limited data point of view, most of the patients (11/15) with uterus didelphys had endometrial cancer on one side of the uterus, two cases had atypical hyperplasia of endometrium on the contralateral uterus. Atypical hyperplasia of endometrium is the precancerous lesion of endometrial adenocarcinoma. 29% of complex atypical hyperplasia without treatment will develop into cancer [23]. Advanced endometrial carcinoma was more common in bilateral uterus, only one case of advanced endometrial cancer occurred in the left uterine cavity with the differentiation type as G3 [14].

Back to our case, the patients was diagnosed as stage IA of moderately differentiated endometrial carcinoma in the left uterus. No cancer or precancerous lesions were detected in the contralateral uterus given the previous hysteroscopy results. Only the affected side of the uterus and appendix were removed in the surgery. It is certain that the patient needs reviewing transvaginal ultrasound, MRI, and hysteroscopy of the reserved uterus in the follow-up treatment.

In order to ascertain the safety of preserving the fertility of this patient, we would like to search for prognostic biomarkers to predict the progression amongst the normal endometrium, endometrial atypical hyperplasia and endometrial cancer. PTEN is the most commonly investigated biomarker in endometrial cancer. However, PTEN is expressed normally in proliferative endometrial glands and stroma but expressed decreasingly in secretory cycle which may results in variable staining [24], Recently, scientists put more interests on PAX2 which is less frequently lost in both normal proliferative and secretory endometrium than PTEN [25].

PAX2 can be expressed by immunohistochemistry inside normal endometrial glandular cells’ nucleus. In 2018, Emma found a progressive decrease in PAX2 expression from proliferative endometrium to endometrial intraepithelial neoplasia (EIN) to endometrial endometrioid carcinoma (EEC) which is also observed in previous studies. It is acknowledged that it can take 30–40 years from normal endometrium to endometrial cancer. Despite of the small sample size, this study is quite popular since its long follow-up of these patients. In Emma’s study, they presented a method and threshold with PAX2 expression H-score as 75. EIN lesions with a PAX2 expression H-score below or equal to 75 (high risk) had significantly lower progression-free survival than those with an H-score above 75 (low risk). Furthermore, more EIN patients with high-risk PAX2 showed progression (73%; 8/11) compared to low-risk patients (8%; 2/25) [26].

In this study, our patient has done hysteroscopic surgeries 4 times in Peking University First Hospital among which the right uterine endometrium was examined 3 times. To better understand the risk of whether the reserved side of uterus will have pathological progress, our pathologist examined PTEN and PAX2 on three right endometrial pathological sections of the patient. The results showed that the right endometrium obtained from three hysteroscopic operations showed strong positive expression of PTEN and PAX2. This may tell clinicians that the possibility of endometrial lesions on the right side is not significant from the onset to the last operation. In the follow-up process, the PTEN and PAX2 immunohistochemical analysis of endometrium can be carried out. If possible, the H score of PAX2 can be used to better complete the follow-up of endometrial lesions, so as to adjust the treatment plan in time.

Endometrial cancer occurring in patients with uterus didelphys is quite rare in published articles, especially in young patients with needs for childbearing. In this case report, the patient has the unilateral uterus with endometrial cancer removed due to the unresponsiveness to drugs. According to the IHC analysis of the reserved side uterine endometrium, there is no pathological progress until the last hysteroscopic surgery. However, with less evidence, it requires more follow-up in later period to trace the patient’s tumor and fertility outcome. In order to shorten the time required for fertility, the patient chose assisted reproductive technology in our hospital. During the process of ovarian hyperstimulation and After the achievement of fertility outcome, we will keep monitoring the patient’s transvaginal ultrasound, MRI, and hysteroscopy. The application of H score of PAX2 will be considered to evaluate the risk of reserving the contralateral uterus.

## Data Availability

Data sharing is not applicable to this article as no datasets were generated or analyzed during the current study.

## Abbreviations

IHC: Immunohistochemistry
ER: Estrogen receptor
PR: Progesterone receptor
MRI: Magnetic resonance imaging
MMR: Mismatch repair
PTEN: Phosphatase and Tensin homolog deleted on chromosome ten
PAX2: Paired box gene 2
EIN: Endometrial intraepithelial neoplasia
EEC: Endometrial endometrioid carcinoma
